# Altered Phase Separation and Cellular Impact in *C9orf72*-Linked ALS/FTD

**DOI:** 10.3389/fncel.2021.664151

**Published:** 2021-04-21

**Authors:** Daniel A. Solomon, Rebekah Smikle, Matthew J. Reid, Sarah Mizielinska

**Affiliations:** ^1^UK Dementia Research Institute at King’s College London, London, United Kingdom; ^2^Department of Basic and Clinical Neuroscience, Institute of Psychiatry, Psychology and Neuroscience, King’s College London, Maurice Wohl Clinical Neuroscience Institute, London, United Kingdom

**Keywords:** phase separation, *C9orf72*, membraneless organelles, TDP-43, therapeutics

## Abstract

Since the discovery of the *C9orf72* repeat expansion mutation as causative for chromosome 9-linked amyotrophic lateral sclerosis (ALS) and frontotemporal dementia (FTD) in 2011, a multitude of cellular pathways have been implicated. However, evidence has also been accumulating for a key mechanism of cellular compartmentalization—phase separation. Liquid-liquid phase separation (LLPS) is fundamental for the formation of membraneless organelles including stress granules, the nucleolus, Cajal bodies, nuclear speckles and the central channel of the nuclear pore. Evidence has now accumulated showing that the formation and function of these membraneless organelles is impaired by both the toxic arginine rich dipeptide repeat proteins (DPRs), translated from the *C9orf72* repeat RNA transcript, and the repeat RNA itself. Both the arginine rich DPRs and repeat RNA themselves undergo phase separation and disrupt the physiological phase separation of proteins involved in the formation of these liquid-like organelles. Hence abnormal phase separation may explain a number of pathological cellular phenomena associated with *C9orf72*-ALS/FTD. In this review article, we will discuss the principles of phase separation, phase separation of the DPRs and repeat RNA themselves and how they perturb LLPS associated with membraneless organelles and the functional consequences of this. We will then discuss how phase separation may impact the major pathological feature of *C9orf72*-ALS/FTD, TDP-43 proteinopathy, and how LLPS may be targeted therapeutically in disease.

## Introduction

The *C9orf72* mutation is an expansion of a GGGGCC (G_4_C_2_) repeat in intron 1 of the gene. In unaffected individuals the G_4_C_2_ is repeated 2 to 23 times, whereas in those with the mutation, the sequence is expanded to contain hundreds to thousands of repeats (DeJesus-Hernandez et al., 2011; Renton et al., 2011). Due to its location upstream of the coding region, the mutation can lead to a reduction in the levels of the protein that it encodes (Xiao et al., 2015; Sivadasan et al., 2016), which is involved in the regulation of endo-lysosomal trafficking and autophagy (Farg et al., 2014; Sellier et al., 2016; Webster et al., 2016). However, a common finding from murine *C9orf72* knockout models is the lack of neurodegeneration or TDP-43 pathology—a key pathological feature of *C9orf72*-ALS/FTD (O’Rourke et al., 2015; Atanasio et al., 2016; Burberry et al., 2016; Jiang et al., 2016; Sudria-Lopez et al., 2016; Sullivan et al., 2016). These findings indicate that loss of protein function is not sufficient to cause the disease. However, effects of reduced *C9orf72* in autophagy, immune dysregulation (O’Rourke et al., 2015; Atanasio et al., 2016; Burberry et al., 2016; Jiang et al., 2016; Sellier et al., 2016; Sudria-Lopez et al., 2016; Sullivan et al., 2016; Ugolino et al., 2016; Webster et al., 2016; Shi et al., 2018; Zhu et al., 2020), axon growth (Sivadasan et al., 2016) and stress granule dynamics (Maharjan et al., 2017) propose a modulatory role in disease pathogenesis.

However, similar to other non-coding repeat expansion mutations, the G_4_C_2_ repeat produces repetitive RNA which is translated into repetitive polypeptides, which have both been proposed to cause pathogenesis (Mizielinska and Isaacs, 2014). The G_4_C_2_ repeat is transcribed in both directions generating G_4_C_2_ (sense) and C_4_G_2_ (antisense) repeat RNA. These RNA species both form small RNA aggregates called RNA foci in patient brain (DeJesus-Hernandez et al., 2011; Gendron et al., 2013; Lagier-Tourenne et al., 2013; Mizielinska et al., 2013; Mackenzie et al., 2014; DeJesus-Hernandez et al., 2017), which may cause dysfunction by sequestering RNA-binding proteins (Almeida et al., 2013; Donnelly et al., 2013; Lee et al., 2013; Sareen et al., 2013; Cooper-Knock et al., 2014, 2015; Haeusler et al., 2014; Rossi et al., 2015; Mori et al., 2016; Celona et al., 2017). Both sense and antisense repeat RNA also undergo non ATG-dependent translation into repetitive polypeptides. The polypeptides produced consist of two alternating amino acids (due to the repetitive RNA code) and are thus termed dipeptide repeat proteins (DPRs). Translation occurs in all six possible frames, three sense and three antisense, producing five different DPRs as one sense and antisense frame are the same. These are polypeptides of glycine-proline (poly-GP), glycine-alanine (poly-GA), glycine-arginine (poly-GR), proline-arginine (poly-PR) and proline-alanine (poly-PA). These DPRs are also all found to form inclusions in patient brain (Ash et al., 2013; Mori et al., 2013a), but seem largely distinct from the classic TDP-43 pathology. *In vitro* and *in vivo* models show that the arginine rich DPRs poly-GR and poly-PR and the most aggregation prone DPR poly-GA can induce significant toxicity (Kwon et al., 2014; May et al., 2014; Mizielinska et al., 2014; Wen et al., 2014; Zhang et al., 2014, 2019a; Tao et al., 2015; Lee et al., 2016; Schludi et al., 2017; Choi et al., 2019; Hao et al., 2019; Cook et al., 2020; LaClair et al., 2020; Zhou et al., 2020). *In vivo* studies have also suggested a direct role for the repeat RNA, albeit with associations with cytoplasmic RNA rather than the classic nuclear foci (Burguete et al., 2015; Swinnen et al., 2018).

A number of cellular processes have been shown to be impaired by the repeat RNA and/or the arginine rich DPRs including the regulation of transcription, ribosomal biogenesis and translation, nucleocytoplasmic transport and RNA granules (Balendra and Isaacs, 2018; Mandrioli et al., 2020). All of these cellular processes are associated with membraneless organelles—multicomponent, viscous liquid-like structures that lack a lipid bilayer. These membraneless assemblies are found in both the nucleus and cytoplasm and typically contain both RNA and protein molecules. Examples of such membraneless organelles include the nucleolus, nuclear pore complex, stress granules, nuclear speckles, paraspeckles, p-bodies and Cajal bodies (Brangwynne et al., 2015; Freibaum and Taylor, 2017). The altered assembly, dynamics, and function of membraneless organelles may account for many of the widespread cellular abnormalities observed in *C9orf72*-ALS/FTD and can explain several of the mechanisms associated with both G_4_C_2_ repeat RNA and arginine rich DPR toxicity.

## Protein Phase Separation

**Figure 1:**
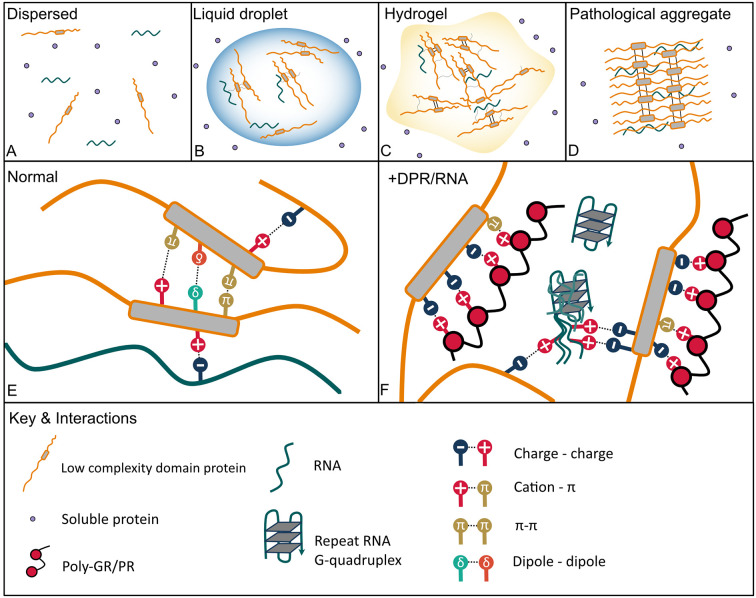
Protein phase transition states and interactions with *C9orf72* arginine rich dipeptide repeat proteins (DPRs) and GGGGCC (G_4_C_2_) repeat RNA that lead to aberrant phase separation. **(A)** Dispersed—soluble proteins are freely dispersed in a dilute phase. **(B)** Proteins with intrinsically disordered regions such as low complexity domains (LCDs) can de-mix from a dilute phase often in the presence of RNA through liquid-liquid phase separation (LLPS) to form dynamic liquid droplets in which molecules can still diffuse in and out and disperse rapidly. **(C)** Liquid droplets can transition into more solid-like hydrogels which consist of amyloid-like fibrils, which are less dynamic but still reversable. **(D)** Aberrant phase separation facilitates the formation of pathological permanent amyloid fibrils which are the constituents of aggregates found in neurodegenerative disease brain. **(E)** Physiological interactions that mediate LLPS include pi (Π)–Π, cation–Π, polar (dipole-dipole), charge-charge interactions in addition to hydrophobic and Π-sp2 interactions, and hydrogen bonding (not shown). **(F)** The arginine-rich DPRs mimic physiological interactions driving aberrant phase separation with the arginine residues acting as the cation in electrostatic interactions, both charge-charge and with aromatic side chains within LCDs (cation-Π). The G_4_C_2_ repeat RNA can also undergo charge-charge interactions with LCDs; further G_4_C_2_ G-quadruplexes may enhance RNA-RNA and RNA-LCD interactions and nucleate droplet formation by cellular RNA.

In cell biology liquid-liquid phase separation (LLPS) is a process in which a homogenous liquid solution consisting of RNA and/or protein separates into two different phases; with one of these separated phases containing an increased concentration of RNA and protein—the dense phase, and the other phase—known as the dilute phase—now depleted of them (Alberti and Dormann, 2019; Figures 1A,B). The dense phase usually resembles liquid droplets and indeed shows liquid-like properties including fusion, coalescence, dripping and a rapid exchange of molecules (Alberti and Dormann, 2019; Alberti et al., 2019). This phase separation of organic molecules into droplets through LLPS is known as coacervation and the resulting droplets can also be referred to as coacervates (Deshpande et al., 2019). Membraneless organelle formation most frequently occurs through spontaneous LLPS in which protein laden RNAs separate themselves from the surrounding aqueous nucleoplasm or cytoplasm forming a reversible state which can equally quickly dissolve (Brangwynne et al., 2015; Taylor et al., 2016).

The formation of membraneless organelles has been described as a dynamic liquid demixing process in which cells actively generate phase boundaries to confine functional entities for a temporary period (Aguzzi and Altmeyer, 2016). Once LLPS has occurred it is thought the proteins within the dense phase compartment find themselves in a different solvent environment compared to their surroundings, which may promote specific biochemical interactions and thus functionalities (Aguzzi and Altmeyer, 2016). Most of the proteins that drive intracellular phase separation and the formation of membraneless organelles show strong conformational heterogeneity and are referred to together as intrinsically disordered proteins (Brangwynne et al., 2015; Boeynaems et al., 2018). These proteins do not have well defined protein folding and are highly flexible due to significantly reduced numbers of aliphatic and aromatic residues (Alberti and Dormann, 2019). However, many of these proteins still contain structured regions and only segments that do not form a well-defined tertiary three-dimensional structure; these disordered regions of the protein are referred to as intrinsically disordered regions. The structural plasticity of these disordered proteins and regions allows them to dynamically adopt different confirmations such as energetically favorable higher-order protein assemblies and undergo a multitude of promiscuous multivalent interactions (Aguzzi and Altmeyer, 2016). Such factors dictate the behavior of disordered proteins within complex liquids like the intracellular milieu and facilitate phase separation (Hyman et al., 2014; Brangwynne et al., 2015; Aguzzi and Altmeyer, 2016; Boeynaems et al., 2018). Thus, these multivalent interactions are important for the formation of dynamic heterogeneous assembles, such as membraneless organelles (Mitrea and Kriwacki, 2016; Freibaum and Taylor, 2017).

Intrinsically disordered regions typically contain repetitive sequence strings biased towards specific amino acids (Boeynaems et al., 2018) and are categorized based upon the composition of their sequence and motifs (Alberti and Dormann, 2019). One important example is low complexity sequence domains (LCD)—amino acid sequences between 75 and 300 amino acids in length with a high evolutionary conservation, present in one-third of the human proteome (Freibaum and Taylor, 2017). These domains often consist of a high number of uncharged polar amino acids such as glycine, asparagine and serine interspersed with aromatic and charged residues (Wang et al., 2018b). The low sequence diversity generates multiple short motifs and repeats such as, glycine/serine-phenylalanine/tyrosine-glycine/serine, arginine-glycine, phenylalanine-glycine, poly-glutamine, poly-asparagine, and blocks of positive or negative charges important for the formation of ribonucleoprotein granules and other biomolecular condensates (Banani et al., 2017; Feng et al., 2019). Specific LCDs such as prion-like domains and glycine-arginine rich (RGG) domains found in RNA-binding proteins are also named for their composition patterns with prion-like domains enriched in glutamine-asparagine and aromatic residues, similar to yeast prion sequences. The residues within these domains mediate several important interactions that mediate phase separation including charge-charge, cation–pi, pi–pi, and polar (dipole-dipole) in addition to hydrophobic and pi/sp^2^ interactions, and hydrogen bonding (Murthy et al., 2019; Peran and Mittag, 2020; Figure 1E). Pi (Π)-stacking interactions occur between delocalized pi electrons in aromatic rings but also between planar non-aromatic residues, such as arginine, glutamine, asparagine, aspartic acid and glutamic acid (Vernon et al., 2018). Positively charged residues, most commonly arginine, can also form cation-pi interactions with electron-rich aromatic residues. Oppositely charged residues arranged in like-charged clusters also undergo charge neutralization by electrostatic charge-charge interactions. These side-chain interactions have been shown to mediate LCD phase separation in a number of condensate biomolecules (Feng et al., 2019; Spannl et al., 2019) and post-translational modifications, missense mutations or scrambling charge clustering inhibits droplet formation by disrupting relevant weak interactions (Feng et al., 2019). These multivalent, low affinity associations mediate interactions that can be rapidly rearranged, including both protein-protein and protein-nucleic acid interactions (Aguzzi and Altmeyer, 2016; Boeynaems et al., 2018; Alberti and Dormann, 2019). Indeed, RNA plays a key role in the formation of the majority of cellular membraneless organelles *via* phase separation acting as a scaffold for LCD interactions and thus RNA concentration can determine the phase separation behavior of LCD proteins depending on their subcellular location (Langdon and Gladfelter, 2018; Maharana et al., 2018; Garcia-Jove Navarro et al., 2019; Rhine et al., 2020). It is suggested that the ability of RNA to undergo numerous multivalent interactions along with its flexible structure means that RNA essentially imitates the LCD of proteins (Rhine et al., 2020). Further RNA itself has been shown to phase separate *via* RNA-RNA interactions mediated by electrostatic forces (Jain and Vale, 2017).

Some dense protein solutions have the potential to further phase transition into structures with properties resembling a solid—a process that has been referred to as gelation (Alberti and Dormann, 2019; Figure 1C). Indeed, whilst many ribonucleoprotein granules are liquid like, other membraneless organelles feature solid-like properties, such as the central channel of the nuclear pore (Frey et al., 2006; Schmidt and Görlich, 2015). Indeed, numerous RNA binding proteins, including FUS and hnRNPA1, transition into reversable hydrogels composed of amyloid-like cross-β fibrils (Kato et al., 2012; Molliex et al., 2015; Murray et al., 2017; Gui et al., 2019). These fibrils however differ from the highly stable amyloid fibrils seen in pathological aggregates formed by proteins associated with neurodegenerative disease (Figure 1D) as the amyloid formation associated with hydrogel formation of LCDs is reversable (Murray et al., 2017; Gui et al., 2019). This reversibility is associated with short motifs that allow amyloid-like β-strand interactions known as kinked β sheets, termed LARKs (low-complexity aromatic-rich kinked sequences). LARKs form weakly stabilizing fibrils (Gomes and Shorter, 2019) unlike cross-β sheets of amyloid fibrils which form stable pathogenic steric “zippers” due to interdigitated side chain amino acids. LCDs of RNA binding proteins that undergo LLPS to form membraneless organelles are enriched in LARKs, suggesting a possible role in LLPS (Hughes et al., 2018). Further the hydrogel forming phenylalanine-glycine domain of nuclear pore proteins is also thought to form LARKs (Hughes et al., 2018). Other motifs that form reversible amyloid fibrils have also been identified in the LCDs of FUS, hnRNPA1 and hnRNPA2 (Luo et al., 2018; Gui et al., 2019; Lu et al., 2020; Sun et al., 2020b). Hence amyloid formation may have a practical function in membraneless organelle formation, for more stable, less transient structures. However, this acquisition of solid-like properties has also been associated with the formation of protein aggregates in neurodegenerative disease (Elbaum-Garfinkle, 2019). Disease mutations in the LCD of proteins have been shown to alter phase separation behavior, generally promoting phase separation and reducing droplet dynamics: ALS associated mutations in FUS lead to the formation of more solid-like droplets and irreversible fibrillar hydrogels which can trap other RNA binding proteins (Murakami et al., 2015; Patel et al., 2015). Similarly, phase separation of hnRNPA1 with the disease mutation D262V causes enhanced amyloid fibril formation (Lin et al., 2015). ALS causing mutations in TIA1 lead to phase separated droplets with reduced mobility and faster fibrilization (Mackenzie et al., 2017). Finally, mutations in the C-terminal domain of TDP-43 enhance its phase separation and show reduced droplet fluidity (Conicella et al., 2020). Thus, phase separation underlies the localization and function of many LCD containing proteins, and disruption in the physiological equilibrium of this process can lead to pathological behavior of the membraneless organelles they are associated with.

### *C9orf72* DPR Phase Separation

In relation to phase separation, there is particular interest in the arginine rich *C9orf72* DPRs due to the well-established role of intrinsically disordered arginine rich regions as promoters of phase separation. Arginine and glycine rich motifs (RGG/RG) are an important class of LCD sequence found within numerous RNA binding proteins that undergo LLPS, including those associated with neurodegenerative diseases such as FUS, hnRNPA1, and FMRP (Thandapani et al., 2013; Chong et al., 2018). Proteins with these motifs contain varying numbers of RGG/RG repeats interspaced typically with aromatic residues (Thandapani et al., 2013), with repeat length influencing multivalency and phase separation (Chong et al., 2018); phase separation of these domains also correlates with the number of arginine residues present (Chong et al., 2018; Wang et al., 2018b). Within FUS, interactions between aromatic residues in the prion-like domain and positively charged residues in the RNA binding domain determine the saturation concentration, which appeared specific to tyrosine-arginine interactions (Wang et al., 2018b). Increasing the negative charge of the prion-like domain increased phase separation mediated by interaction with the RNA binding domain, but reduced phase separation in isolation. Hence electrostatic attractions between arginine residues and negatively charged residues in the prion-like domain additionally regulate phase separation by bolstering the interaction between aromatic tyrosine residues and positively charged arginines in the RNA binding domain, whereas electrostatic repulsions within the prion-like domain help prevent unfruitful self-interactions. This is reflected in the toxicity of FUS in HeLa cells, where the severity of FUS toxicity correlates with the strength of the interaction between the prion-like domain and RNA binding domain, modulated by arginine residues in the RNA binding domain (Wang et al., 2018b). Functionally, the arginine residues in the C-terminal RNA binding domain of FUS are crucial for the maturation of the protein in stress granules, recruitment to sites of DNA damage and also regulate its toxicity in *Drosophila* (Bogaert et al., 2018).

The *C9orf72* arginine rich DPRs poly-GR and poly-PR, translated from the G_4_C_2_ repeat expansion, undergo phase separation (in the presence of a crowding agent) *in vitro* similarly to RGG/RG domains (Boeynaems et al., 2017). Further analysis of the phase separation of poly-PR revealed that the droplets formed have liquid-like properties—with the droplets being circular in shape, showing recovery upon photobleaching (due to fast internal rearrangement) and deformation under stress, fusing together and being reversible upon dilution or changes in temperature or solute. Poly-PR phase separation was also impaired by elevating salt concentration, indicating that arginine-driven electrostatic forces modulate poly-PR LLPS. Normally, the coacervation of molecules into droplets is inhibited when proteins consist primarily of a single charge due to charge repulsion and require the presence of counter ions (Pak et al., 2016; Boeynaems et al., 2017). Indeed, phase separation of poly-GR and poly-PR does not result from electrostatic repulsion between the arginine residues of the dipeptide, but rather due to anions in the buffer, with anions with more valence driving droplet formation whereas monovalent anions which exist in a single charge state being inhibitory (Boeynaems et al., 2017; Jafarinia et al., 2020). Biological polyvalent anions such as poly-uracil RNA can even overcome the need for crowding agents in poly-PR LLPS (Boeynaems et al., 2017). Furthermore, like arginine residues in the RNA binding domain of FUS, poly-PR also engages in cation-pi interactions with tyrosine. Therefore, the phase separation of the arginine rich DPRs poly-GR and poly-PR and likely their interactions with other molecules that contain LCDs are similar to that of arginine rich RNA binding proteins like FUS: complex coacervation driven by electrostatic interactions, both charge-charge and charge-pi, with the arginine residues providing the cation for these weak, multivalent interactions (Nedelsky and Taylor, 2019; Figure 1F).

Further analysis of poly-PR phase separation *via* complex coacervation revealed that the process is also governed by the chemistry of associated polyanions (Boeynaems et al., 2019). Negatively charged protein assemblies such as microtubules provide a scaffold for poly-PR recruitment whereas flexible polyanions such as RNA caused spherical droplet formation. The latter is in line with LLPS *via* complex coacervation with the dynamics of droplets varying depending on the polyanion used. Poly-PR-RNA LLPS is dependent on the structure of the RNA; all homopolymeric RNAs, except for poly-rGuanine lead to poly-PR liquid droplet formation. The properties of these droplets, such as viscosity, are dependent on the specific RNA molecules involved in RNA-PR and RNA-RNA interactions. The lack of coacervation of poly-PR in presence of poly-rGuanine is connected to higher-order RNA structures formed by poly-rGuanine. Poly-rGuanine unlike the other RNA bases can form a highly stable secondary structure known as a G-quadruplex, which appears to kinetically stall poly-PR phase separation. Boeynaems et al. (2019) interpret this as poly-PR being unable to outcompete base stacking interactions within G-quadruplexes. The length of the DPR in addition to the length of its interacting polyanion also influences droplet size due to increased multivalency; with modeling showing that larger PR molecules and larger polyanions produce smaller droplets of a higher concentration (Jafarinia et al., 2020). Hence variations in the size of droplets owe to variations in the robustness of poly-PR interactions and the molecules it is phase separating with. Similar findings have been observed for poly-GR post-translational modifications; poly-GR aggregates have been found to be methylated in patient brain and synthetic dimethylated poly-GR peptides form larger but reduced numbers of droplets compared to unmethylated poly-GR, indicative of reduced LLPS (Gittings et al., 2020). Arginine methylation does not disrupt charge but may affect cation-pi interactions, as it does when disrupting the LLPS of several RNA-binding proteins (Qamar et al., 2018; Ryan et al., 2018; Hofweber and Dormann, 2019), and thereby would be expected to weaken pathological interactions of the arginine rich DPRs and LCD containing proteins.

One question that arises from these studies is, whether the ability to undergo phase separation is what causes the severe toxicity of poly-PR and poly-GR. When peptides of different composition were studied, expression of a poly-arginine only peptide located to the cytoplasm and showed no toxicity (Meloni et al., 2013, 2015, 2017) whereas PR_12_ with the same number of arginine residues was highly toxic and localized to the nucleus (Kanekura et al., 2018). Indeed poly-PR peptides and constructs in model systems typically show higher toxicity than poly-GR of similar lengths and number of arginine residues (Wen et al., 2014). Hence arginine content and the ability to undergo LLPS are not the only determining factors in the toxicity of these DPRs. One theory of FUS separation proposed by Wang et al. (2018b) uses the concept of stickers and spacers, in which the stickers (such as arginine) determine the phase separation properties of the protein; and the spacers (such as glycine or proline) which separate the stickers determine the flexibility of the peptide. The different spacers found in poly-GR (glycine) and poly-PR (proline) may influence the interactions undergone by the peptides thereby influencing their relative toxicities. Additionally, the methylation of poly-GR reduces its propensity for LLPS and decreases toxicity in neurons, and symmetric dimethylation of poly-GR inclusions in *C9orf72*-FTD/ALS patient brain positively correlates with disease duration, suggesting methylation of poly-GR and reduced LLPS could be protective (Gittings et al., 2020).

### *C9orf72* Repeat RNA Phase Separation

As already mentioned, RNA plays an important regulatory role in driving phase separation of intrinsically disordered proteins and the physical properties of resultant droplets, dependent on concentration, secondary structure and sequence of RNA (Langdon and Gladfelter, 2018; Zhang et al., 2019b). The guanine (G)-rich DNA and RNA of the *C9orf72* repeat expansion forms highly complex secondary structures including unimolecular and multimolecular G-quadruplexes (Fratta et al., 2012; Reddy et al., 2013; Haeusler et al., 2014; Zhou et al., 2015; Conlon et al., 2016). G-quadruplexes are stable four strand structures formed of planar guanine tetramers stacked on top of one another (Zhou et al., 2015). G-quadruplex containing RNA has been reported to itself undergo phase separation (Zhang et al., 2019b), as has triplet repeat RNAs of CAG and CUG (Jain and Vale, 2017). Indeed, similarly to CAG and CUG repeat RNA, *C9orf72* G_4_C_2_, but not antisense C_4_G_2_, repeat RNA directly formed gels *in vitro* in a repeat-length dependent manner and was disrupted by monovalent cations and antisense oligonucleotides (ASOs), indicating both electrostatic interactions and base-pairing interactions (Jain and Vale, 2017; Figure 1F). Interestingly, the authors speculate that the increased valency that comes with repeat length may explain the length-dependent threshold that exists in disease (Langbehn et al., 2010; Rohrer et al., 2015). In cells, CAG repeat RNA formed nuclear foci that had liquid properties, which was proposed to be due to the presence of RNA-binding proteins such as helicases which remodel RNA-base pairing. Whereas, G_4_C_2_ RNA foci showed only partial recovery after photobleaching, indicating that they are less dynamic than CAG foci. In another study, G_4_C_2_ repeat RNA LLPS only occurred in the presence of cell lysate and required cellular RNA but was equally dependent on repeat-length and electrostatic interactions (Fay et al., 2017). In this study, G_4_C_2_ RNA precipitated stress granule proteins, and thus will be discussed in the relevant section below.

## Disrupted Phase Separation of Membraneless Organelles

**Figure 2:**
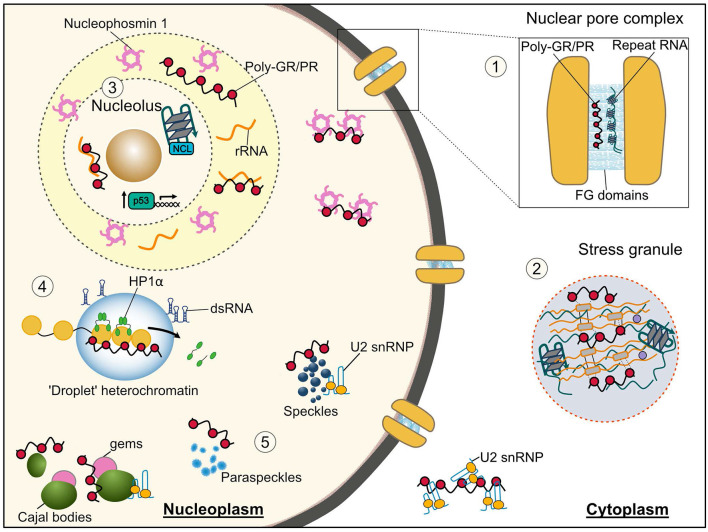
Membraneless organelles and associated functions that are impaired by the *C9orf72* arginine rich DPRs and G_4_C_2_ repeat RNA. **(1)** Nuclear pore complex—Phenylalanine-glycine low complexity domains (LCDs) of nuclear pore proteins (nucleoporins) phase separate into a selective hydrogel in the central channel of the nuclear pore. Both the arginine rich DPRs and G_4_C_2_ RNA have been shown to interact with these nucleoporins, thus their altered phase separation may underlie the nucleocytoplasmic transport dysfunction seen in *C9orf72*-ALS/FTD. **(2)** Stress granules—both the arginine rich DPRs and repeat RNA bind and cause the aberrant phase separation of LCD containing stress granule proteins inducing formation of poorly dynamic stress granules, which likely contributes to the translational repression seen in *C9orf72*-ALS/FTD models. **(3)** Nucleolus—Poly-PR disrupts the phase separation of the nucleolar protein nucleophosmin with ribosomal RNA leading to its mislocalization from nucleoli to the nucleoplasm, and an impairment in ribosomal biogenesis; this nucleolar stress may also lead to the observed aberrant activation of the p53 pathway. The G-quadruplex forming G_4_C_2_ RNA has also been shown to associate with the nucleolar protein nucleolin (NCL) and cause nucleolar dysfunction. **(4)** Heterochromatin—poly-PR perturbs phase separation of heterochromatin protein 1α (HP1α) displacing it from heterochromatin and resulting in its degradation and an upregulation of repetitive RNA elements which form double-stranded RNA and initiate pathological interferon signaling. **(5)** Nuclear structures—proteins associated with nuclear speckles, paraspeckles, Cajal bodies and Gems interact with both the arginine rich DPRs and G_4_C_2_ RNA. The arginine rich DPRs have specifically been shown alter the liquid-like properties of these organelles and binding to LCD containing U2 snRNP proteins results in their mislocalization from nuclear speckles to the cytosol and reduced splicing activity.

As already mentioned, phase separation is key to the formation of membraneless organelles, including stress granules, the nucleolus, Cajal bodies, nuclear speckles, and the central channel of the nuclear pore. These liquid organelles regulate several important cellular functions, include splicing, protein translation, nucleocytoplasmic transport and the cellular stress response. A disruption in any one of these molecular functions is likely to be catastrophic for a cell. In the following sections, we review the evidence that the *C9orf72* arginine rich DPRs and G_4_C_2_ RNA disrupt the phase separation of these membraneless organelles and the cellular pathways associated with them (Figure 2).

### Nuclear Structures

In the nucleus, there are a variety of different membraneless structures formed by LLPS including nuclear speckles, paraspeckles, Cajal bodies and Gems. These are multiprotein-RNA organelles that play an essential role in transcriptional regulation and the formation and function of the spliceosome, a large ribonucleoprotein complex where pre-mRNA splicing is catalyzed (Lamond and Spector, 2003; Will and Lührmann, 2011). Cajal bodies are organelles whose major function is the modification and assembly of uridine-rich small nuclear ribonucleoproteins (U snRNPs; Morris, 2008), and Gems (Gemini of Cajal bodies) localize adjacent to Cajal bodies and are characterized by the presence of the SMN (survival of motor neuron) protein (Liu and Dreyfuss, 1996). Mature U snRNPs accumulate in nuclear speckles, also known as splicing speckles, which function as the site for the storage and modification of pre-mRNA splicing factors and pre-mRNA splicing itself (Spector and Lamond, 2011; Galganski et al., 2017; Gruss et al., 2017). Paraspeckles primarily regulate gene expression through sequestration of RNAs and proteins (Fox et al., 2018).

#### DPRs

Nuclear speckles and Cajal body proteins have been shown to interact with the arginine rich DPRs poly-PR and poly-GR, and speckle proteins can modify their toxicity (Lee et al., 2016; Boeynaems et al., 2017; Yin et al., 2017; Hartmann et al., 2018; Moens et al., 2019). The nuclear speckle protein SRSF7 is specifically affected by overexpression of poly-PR but not GR, displaying reduced recovery after photobleaching (Lee et al., 2016), an indication of perturbed LLPS. Poly-PR can also increase the levels of nuclear paraspeckles by direct interaction with paraspeckle proteins and RNA (Suzuki et al., 2018, 2019). In cells, expression of poly-PR, GR or GA leads to a dramatic reduction in Cajal bodies and Gems (Lee et al., 2016; Rossi et al., 2020); a few poly-GR expressing cells displayed increased numbers but were noticeably smaller than controls, indicating that the phase separation properties of Cajal bodies may also be perturbed. Functionally, these changes may explain the changes in gene expression and splicing-associated with expression of both poly-PR and poly-GR (Kwon et al., 2014; Yin et al., 2017; Kramer et al., 2018; Sun et al., 2020a). Relating to Cajal body function, in a proteomic interaction study of poly-GR and poly-PR all known U2 snRNP proteins were found, with six of these as top interactors (Yin et al., 2017). Interestingly, this was specific to the U2 snRNP complex, as only three of eight U5 snRNP components were identified. Poly-GR was further found to inhibit assembly of the spliceosome, a key function of the U2 snRNP complex, and lead to downstream mis-splicing. Perturbation in the U2 snRNP has also been observed in patient cells. Using *C9orf72* iPSC-derived motor neurons which have been shown to produce detectable levels of poly-GR (Lopez-Gonzalez et al., 2016) the U2 snRNP associated proteins SNRPB2 and S3Fa were mislocalized to the cytoplasm, with SNRPB2 mislocalization in around 40–60% of neurons, whereas it was completely nuclear in controls (Yin et al., 2017). This was also recapitulated in cell lines exposed to poly-PR peptide, confirming the link to the DPR. Furthermore, in patient cells with cytoplasmic accumulation of SNRPB2, levels of the protein in nuclear speckles and the nucleoplasm were decreased. Again, this was a specific effect as U1 snRNP proteins were unaffected. This was also associated with a preferential mis-splicing of U2 snRNP dependent exons in patient lines, which is also dominant in patient tissue. The disassembly of Cajal bodies by poly-GR and PR in cells could be rescued by co-expression of the nuclear transport receptor importin β1 (Rossi et al., 2020). As importin β1 mediates, nucleocytoplasmic transport of snRNPs and nucleocytoplasmic transport is impaired during stress (Boeynaems et al., 2016b), the arginine rich DPRs may also impair Cajal body assembly indirectly by disrupting the transport of U2 snRNPs into the nucleus.

#### G_4_C_2_ Repeat RNA

A number of the LCD containing RNA binding proteins that bind to the repeat RNA have important functions in RNA splicing, including the nuclear speckle protein SRSF1 (Hautbergue et al., 2017). SRSF1 is a nuclear speckle protein that facilities pre-mRNA splicing factor assembly and plays a role in nuclear export (Huang et al., 2003; Tripathi et al., 2012). SRSF1 could also be found to colocalize with G_4_C_2_ RNA foci in motor neurons of patient spinal cord sections. Knockdown of SRSF1 can also rescue neurodegeneration in G_4_C_2_ repeat *Drosophila* and reduce toxicity from *C9orf72* patient-derived astrocytes on control motor neurons. Importantly, this rescue was specific to the SRSF1-G_4_C_2_ RNA interaction as SRSF1 knockdown in arginine rich DPR flies resulted in no rescue. Mechanistically, SRSF1 depletion reduced nuclear export of G_4_C_2_ mRNA *via* its interaction with nuclear export receptor NXF1, and subsequent DPR production. Thus, it is proposed that the sequestration of SRSF1 into RNA foci leads to increased nuclear export of G_4_C_2_ RNA and thereby increasing RAN translation, enhancing DPR toxicity in *C9orf72*-ALS/FTD. The effect on the properties of nuclear speckles, however, has not yet been investigated.

### Nucleolus

The nucleolus is a nuclear organelle whose primary function is ribosome biogenesis, including both the synthesis and processing of ribosomal RNA *via* RNA polymerase I and the assembly of ribosomes (Boisvert et al., 2007; Iarovaia et al., 2019). The nucleolus consists of three defined liquid-like phases: the fibrillary centre(s) surrounded by a dense fibrillar component rich in fibrillarin, all encompassed by a granule component enriched in nucleophosmin; the latter is an LCD-containing protein known to undergo phase separation (Mitrea et al., 2018; Frottin et al., 2019).

#### DPRs

An early observation in cell models of the *C9orf72* DPRs poly-GR and poly-PR was a strong nucleolar localization with nucleolar swelling and the displacement of nucleolar proteins to the nucleoplasm (Haeusler et al., 2014; Kwon et al., 2014; May et al., 2014; Wen et al., 2014; Zhang et al., 2014; Tao et al., 2015; Callister et al., 2016; Lee et al., 2016). They have also been shown to directly interact *in vitro* (Lee et al., 2016; Boeynaems et al., 2017; Hartmann et al., 2018). Both poly-GR and poly-PR are recruited to the liquid-like granular component of the nucleolus, and poly-GR could additionally interact with the dense fibrillar components (Lee et al., 2016). Poly-GR and poly-PR peptides facilitate phase separation of nucleophosmin *in vitro*, which is accompanied by a reduction in the mobility of the nucleolar proteins nucleophosmin and nucleolin in the nucleolus of cells expressing either poly-GR or poly-PR (Lee et al., 2016). However, when in molar excess the arginine rich DPRs can inhibit LLPS (Lee et al., 2016; White et al., 2019). They can also outcompete SURF6, an arginine-tract containing protein and native binding partner of nucleophosmin (Lee et al., 2016) and have been found to specifically bind *via* the third acidic tract (A3) within its intrinsically disordered region (White et al., 2019). In this latter study, by disturbing the nucleophosmin-SURF6 interaction which is required for physiological phase separation and causing a dissolution of nucleophosmin particles, poly-PR leads to the sequestration of nucleophosmin into soluble nucleophosmin/poly-PR complexes (White et al., 2019). Poly-PR was also able to outcompete nucleophosmin for ribosomal RNA (rRNA) binding leading to the accumulation of rRNA in rRNA/poly-PR puncta which persisted even at high concentrations of the DPR. These findings were recapitulated in cells where increasing poly-PR concentration caused the delocalization of nucleophosmin from nucleoli into the nucleoplasm and a sequestration of rRNA with poly-PR in the nucleolus; increasing poly-PR peptide length displayed a similar increase in dissolution effect. Hence a disruption of the phase separation of nucleophosmin explains the nucleolar dysfunction observed in cellular and animal models of DPR toxicity.

Functionally, studies have shown that numerous nucleolar functions are perturbed by the arginine rich DPRs such as ribosomal protein transport, the processing of rRNA and the assembly of ribosomes (Kwon et al., 2014; Jovičić et al., 2015; Tao et al., 2015; Kanekura et al., 2018; Suzuki et al., 2018). Co-expression of nucleophosmin can rescue arginine rich DPR toxicity in cells (Farg et al., 2017), and knockout or overexpression of genes encoding proteins in ribosomal rRNA processing can modify poly-PR toxicity in yeast (Jovičić et al., 2015; Chai and Gitler, 2018) and *Drosophila* (Boeynaems et al., 2016a). Notably, homologs of the nucleolar protein nucleolin (NCL), identified in multiple studies, could suppress PR-toxicity upon deletion. In *C9orf72*-ALS/FTD brain tissue, the majority of neuronal nucleoli were smaller in comparison to age-matched controls, however neurons which contained a cytoplasmic poly-GR inclusion had a significantly increased nucleolar volume (Mizielinska et al., 2017). Overexpression of poly-GR (and poly-GA but to a much lesser extent) could also cause nucleolar enlargement in *Drosophila* neurons (Mizielinska et al., 2017), however this has not been recapitulated in mouse models of the arginine rich DPRs (Zhang et al., 2018b, 2019a); these differences have been proposed to differ depending on whether the DPRs expressed can enter the nucleus.

#### G_4_C_2_ Repeat RNA

Although to a lesser extent, nucleolar dysfunction has also been associated with *C9orf72* repeat RNA toxicity. G_4_C_2_ repeat RNA can bind several nucleolar proteins, predominantly in its G-quadruplex form; indeed, NCL could be found in association with RNA foci in the motor cortex of expansion carriers (Haeusler et al., 2014; Cooper-Knock et al., 2015). In cells, this caused a deficit in the production of mature ribosomes and a build-up of untranslated mRNAs in the cytoplasm (Haeusler et al., 2014). A subtle increase in nucleolar volume has also been observed in frontal cortex neurons containing sense RNA foci in *C9orf72*-FTD patient brain (Mizielinska et al., 2017). Interestingly, although *in vitro* the C_4_G_2_ antisense RNA does not bind nucleolar proteins (Haeusler et al., 2014), they are found to more frequently associate and surround the nucleolus in patient tissue (Mizielinska et al., 2013; Vatsavayai et al., 2016; Aladesuyi Arogundade et al., 2019); note, nucleolar proteins do not however associate with antisense RNA foci outside the nucleolus (Cooper-Knock et al., 2015).

### Stress Granules

Stress granules are transient, dynamic, cytoplasmic assemblies which form reversibly under conditions of acute cellular stress, such as heat shock, oxidative stress or nutrient depletion, to sequester non-translating mRNA, translation initiation complexes and related RNA binding proteins. By protecting and temporarily storing stalled translation complexes until stress dissipates, stress granules effectively regulate translation of housekeeping mRNA, while promoting translation of cytoprotective proteins such as chaperones (Baradaran-Heravi et al., 2020). Similarities in the dynamic behavior and liquid-like properties of *in vivo* cellular ribonucleoprotein granules and *in vitro* granule components support the assertion that these compartments form *via* LLPS and interactions between proteins with LCDs and RNA (Hyman et al., 2014; Kroschwald et al., 2015).

#### DPRs

The arginine rich *C9orf72* DPR interactomes are enriched in LCD-containing proteins including RNA-binding proteins and components of stress granules, such as TDP-43, FUS, hnRNPA1, TIA1 and G3BP1 (Lee et al., 2016; Lin et al., 2016; Moens et al., 2019), highlighting a role for disrupted stress granule function in DPR toxicity. *In vitro* poly-PR and GR selectively associate with and decrease the saturation concentration at which droplets form with hnRNPA1, TIA1 and FUS, unlike the non-arginine rich DPRs which have no effect (Lee et al., 2016; Boeynaems et al., 2017). Upon overexpression in cells, poly-GR and PR also increase the formation of stress granules (Lee et al., 2016; Boeynaems et al., 2017). However, the cellular systems vary in their DPR localization with one showing colocalization of stress granules with PR_100_ (Boeynaems et al., 2017) and the other with GR_50_ but not PR_50_ (Lee et al., 2016); this is likely due to the length difference in the PR polypeptides with the shorter version being limited to the nucleus.

Interestingly, numerous disease-linked mutations have been found to enhance LCD polymer stability (Kato and McKnight, 2017; Boeynaems et al., 2018) and a hnRNAP1 variant harboring a mutation which diminishes LCD polymerization (F291S) prevents immunoprecipitation by a PR_20_ peptide (Lin et al., 2016), suggesting that poly-PR interacts with LCDs in a polymeric conformation. PR_30_ treatment also increases the β-sheet content of FUS LCD droplets, as shown by the increase in thioflavin-T fluorescence with increasing poly-PR concentration (Boeynaems et al., 2017). Therefore, DPR interactions with stress granule proteins may increase β-sheet content and enhance stability of stress granule protein LCD polymers, rendering stress granules less dynamic. Indeed, liquid droplets of hnRNPA1, TIA1 or FUS display reduced liquid-like properties when treated with arginine rich DPR peptides, including fewer wetting and fusion events, and reduced recovery from photobleaching (Lee et al., 2016; Boeynaems et al., 2017). This is also recapitulated in cells where arginine rich DPRs induce formation of poorly dynamic stress granules. Live imaging of HeLa cells showed that DPR-induced stress granule G3BP1 puncta increase in number over time and do not appear to disassemble. Photobleaching analysis further confirms that the recovery rate of G3BP1 in DPR-induced stress granules is significantly reduced, compared to arsenite-induced stress granules (Lee et al., 2016; Boeynaems et al., 2017). Poly-PR induced stress granules are also enriched in disease-linked proteins, including ataxin-2 and TDP-43 (Boeynaems et al., 2017), which may reflect their entrapment due to reduced diffusivity or their recruitment may contribute to this process. In mice, the stress granule marker TIA1 remained nuclear and diffuse upon GFP-GR_100_ expression but formed cytoplasmic puncta which colocalized with poly-GR in (G_4_C_2_)_149_ mice using postnatal adenovirus expression (Zhang et al., 2018b). This correlates with the formation of cytoplasmic poly-GR inclusions but not diffuse poly-GR, suggesting that inclusions specifically induce stress granules. Similarly, diffuse GFP-GR_100_ expression in cells did not change the number of stress granules upon heat shock but more stress granules were retained upon recovery, corroborating previous findings in the impairment of disassembly (Zhang et al., 2018b). Thus, arginine rich DPRs nucleate phase separation of LCD-containing stress granule proteins and promote assembly of poorly dynamic stress granules with reduced disassembly compared to adaptive stress granules, highlighting a pathological outcome. The contribution of arginine residues in this process demonstrates that expression of DPRs does not only trigger the stress response, but actively mediates phase separating interactions with stress granule components through high multivalency.

#### G_4_C_2_ Repeat RNA

When G_4_C_2_ RNA is incubated with cellular lysates *in vitro* phase separated particles are formed which are enriched in stress granule components (Fay et al., 2017). Constituents such as G3BP1 and FUS precipitated with all repeat lengths studied, but others including TIA1 only condensed with longer lengths. Particles exhibited classical features of LLPS, including dependence on concentration, temperature, molecular crowding and salt; the latter indicating a role for electrostatic interactions. Phase separation was repeat length dependent and associated with the presence of G-quadruplex forming sequences. Notably, when assays were performed with equal weight of different length G_4_C_2_ repeat RNAs rather than equimolar (where the number of repeat units should be equal) similar condensation was observed, suggesting that repeat number is important, but these can be in *cis* or *trans*. It also required the presence of cellular RNA, suggesting that G-quadruplex G_4_C_2_ RNA enhances intermolecular RNA interactions and thereby promotes the nucleation behavior of RNA that causes protein condensation. Indeed, transfection of G_4_C_2_ RNA in cells leads to the formation of stress granules (Fay et al., 2017). In granules containing both mCherry-tagged G3BP1 and FAM-labeled G_4_C_2_ RNA, photobleaching results in recovery of the G3BP1 signal whereas G_4_C_2_ RNA signal does not. This indicates that within these granules the stress granule protein can rapidly internally rearrange, but the RNA forms a stable component which cannot be replaced. In another study transfection of G_4_C_2_ DNA in cells also induced formation of stress granules, although in this system they did not contain G_4_C_2_ RNA and thus may be induced by the DPRs translated from repeat RNA as detailed above (Rossi et al., 2015).

An important consideration for assessing the relevance of DPRs and RNA to aberrant stress granule phase separation in the wider context of *C9orf72*-ALS/FTD will be evaluating the contribution of each proposed mechanism. Interestingly, the C9orf72 protein has additionally been implicated in stress granule formation (Maharjan et al., 2017). Thus it is likely that there may be interplay amongst mechanisms. Perhaps, loss of C9orf72 protein inhibits the appropriate physiological stress response, then arginine rich DPRs and repeat RNA promote stress granule assembly by nucleating phase separation, and the DPRs subsequently interact with stress granules to reduce dynamics and inhibit disassembly, promoting a state of chronic stress and eventual neurodegeneration.

### The Nuclear Pore

The nuclear pore is a large multiprotein complex comprised of transmembrane nucleoporins that anchor the pore to the nuclear envelope, structural nucleoporins that act as a scaffold and nucleoporins which make up the central channel of the nuclear pore (Cautain et al., 2015). Notably for this review, several nucleoporins contain low complexity phenylalanine-glycine (FG) repeats. These domains primarily localize to the central channel where they form a permeability barrier *via* LLPS critical for nuclear pore selectivity (Frey et al., 2006). *In vitro* these domains spontaneously phase separate into hydrogels that exclude inert macromolecules but allow the entry of nuclear transport receptors, mimicking the selectivity of the nuclear pore in cells (Schmidt and Görlich, 2016).

#### DPRs

DPRs have been associated with defective nucleocytoplasmic transport by the ability of transport factors to modify their toxicity or *via* resultant mislocalization of transport factors in cultured neurons, yeast and *Drosophila* (Jovičić et al., 2015; Boeynaems et al., 2016a; Chai and Gitler, 2018; Solomon et al., 2018). The arginine rich DPRs can also directly interact *in vitro* with different types of transport factors (Lee et al., 2016; Lin et al., 2016; Boeynaems et al., 2017). The arginine rich DPRs bind the low complexity FG domains of some nucleoporins (Lin et al., 2016), being specifically bound when the FG domains are in a polymeric state (Shi et al., 2017). The authors of the latter finding suggest that FG domains of nucleoporins in the nuclear pore exist in equilibrium between polymerized and unpolymerized states, and upon binding poly-PR shifts this equilibrium by stabilizing the polymeric state leading to a less permeable nuclear pore barrier and disruption of transport. The relation of this equilibrium to phase separation has not yet been investigated. Of note, the interaction of the arginine rich DPRs with nucleoporins may not be specific to FG domains, as another study found that binding was only partially reduced when phenylalanines in the FG domain were substituted for alanines (Hayes et al., 2020). Indeed, it has been noted that other low complexity sequences found within the FG domains of nucleoporins (repetitions of the tripeptide sequence glycine/serine-tyrosine-glycine/serine) are similar to the LCDs found in RNA binding proteins (Shi et al., 2017), and thus binding may occur here. Poly-PR (PR_20_) was shown to accumulate in the central channel of the nuclear pore in isolated nuclei from Xenopus oocytes (Shi et al., 2017). However, when GR_200_ was overexpressed using an adenovirus in mice, FG-nucleoporins were found to co-aggregate with poly-GR in the cytoplasm of cortical neurons (Cook et al., 2020). This occurred with concomitant loss of nuclear and cytoplasmic aggregation of TDP-43, indicating that the arginine rich DPR-induced defects of FG-nucleoporins can lead to TDP-43 mislocalization, a key marker of nucleocytoplasmic transport dysfunction and neurodegeneration.

#### G_4_C_2_ Repeat RNA

A recent study has provided evidence that *C9orf72* G_4_C_2_ RNA specifically disrupts the function of the nuclear pore *via* by inducing loss of a specific subset of eight nucleoporins driven by loss of one key nucleoporin POM121 (Coyne et al., 2020). Notably, changes were specifically attributed to G_4_C_2_ RNA and not DPRs as overexpression of either poly-GR or poly-PR were insufficient to induce similar changes, but expression of RNA-only G_4_C_2_ repeats with stop codons inserted to prevent RAN translation were. POM121 is an FG-domain containing nucleoporin that can phase separate into a hydrogel that mimics active and passive nucleocytoplasmic transport (Labokha et al., 2013). Given the ability of the G_4_C_2_ RNA itself to phase separate and promote the phase separation of other LCD containing proteins (Fay et al., 2017; Jain and Vale, 2017), it will be important to study whether G_4_C_2_ RNA can disrupt the phase separation of POM121.

## Discussion

Since the discovery of the *C9orf72* repeat expansion mutation in 2011, major progress has been made in elucidating the underlying pathogenic mechanisms. Both the G_4_C_2_ repeat RNA and DPRs have been shown to be neurotoxic and disrupt a number of cellular processes including nucleocytoplasmic transport, the stress response, nucleolar dysfunction and RNA processing. All these processes require the correct assembly, dynamics, and function of membraneless organelles formed by physiological phase separation of LCD-containing proteins or domains. Both the repeat RNA and arginine rich DPRs themselves undergo phase separation and can disrupt the phase separation of other LCD proteins required for membraneless organelle formation and function. These disturbed phase transitions account for widespread cellular abnormalities observed in *C9orf72*-ALS/FTD and may be a target for therapeutic intervention.

### Phase Separation Underlies Shared Pathomechanisms

In repeat expansion disorders a unique circumstance is produced whereby repetitive RNA and repetitive polypeptides are produced, which due to their repetitive nature are domains of low complexity. This is the most overt in disorders where the expansion is large, as is the case for *C9orf72*-ALS/FTD. Intriguingly, in the evidence highlighted above both the *C9orf72* DPRs and G_4_C_2_ repeat RNA affect many of the same pathways although the RNA and polypeptides have dramatically different structures and thus you would anticipate different interaction partners. It is likely that the arginine rich DPRs result in the convergence of these molecules as the strong positive charge from their arginine content lends them to interact with nucleic acids and their binding partners. The combination of low complexity proteins and RNA is also a key driver of the multivalent interactions that lead to phase separation, present in the majority of the membraneless organelles discussed above. Both the DPRs and repeat RNA may act as molecular seeds initiating aberrant phase transitions by binding to the LCD of intrinsically disordered proteins and causing liquid demixing. Furthermore, for the DPRs, due to the strength of their interactions with LCDs, proteins will no longer interact with their normal binding partners thus disrupting the assembly of membraneless organelles and their physiological function. Hence the majority of observed pathological phenomena and perturbed pathways associated with *C9orf72*-ALS/FTD converge on a disruption of LLPS of membraneless organelles.

#### RNA Splicing

One of these convergences is on RNA splicing. As detailed above, the arginine rich DPRs can bind the LCD containing U2 snRNP proteins leading to their mislocalization from nuclear speckles to the cytosol, an alteration in the liquid-like properties of the nuclear speckles and reduced splicing activity (Lee et al., 2016; Freibaum and Taylor, 2017; Yin et al., 2017). Poly-PR can also increase the levels of nuclear paraspeckles by direct interaction with paraspeckle proteins and RNA (Suzuki et al., 2018, 2019). G_4_C_2_ repeat RNA also binds to the nuclear speckle protein SRSF1, sequestering it into RNA foci and this interaction leads to increased nuclear export of G_4_C_2_ RNA, thereby increasing RAN translation and enhancing DPR toxicity (Hautbergue et al., 2017). The transcriptional regulators Pur-α, a binding partner of SRSF1, and Matrin-3 can also bind G_4_C_2_ repeat RNA and modify toxicity; both are also mislocalized to the cytoplasm upon G_4_C_2_ RNA expression (Xu et al., 2013; Ramesh et al., 2020), with Pur-α being recruited to stress granules (Rossi et al., 2015). Similarly, to SRSF1, Matrin-3 also reduced levels of RAN-translation products (Ramesh et al., 2020). HnRNP H is also a strong interactor of G_4_C_2_ repeat RNA and is sequestered into RNA foci in patients where its depletion results in the reduction in alternative splicing of its targets (Lee et al., 2013; Conlon et al., 2016). The interaction of these DPR and RNA gain of function mechanisms has not yet been studied.

#### Translation

Translation is also targeted. As detailed above, nucleolar function can be impacted by both DPRs and G_4_C_2_ repeat RNA. The arginine rich DPRs bind nucleolar proteins, particularly nucleophosmin, and can both facilitate and inhibit their phase separation depending on DPR concentration; this results in consequent reduced mobility of proteins in the nucleolus, displacement of proteins and ribosomal RNA away from the nucleolus and functional impairment of ribosome biogenesis (Lee et al., 2016; White et al., 2019). Both poly-GR and PR have also been shown to directly bind both cytoplasmic and mitochondrial ribosomal proteins and translation initiation and elongations factors (Kanekura et al., 2016; Lee et al., 2016; Lin et al., 2016; Lopez-Gonzalez et al., 2016; Boeynaems et al., 2017; Yin et al., 2017; Hartmann et al., 2018; Moens et al., 2019; Radwan et al., 2020); the translation initiation factor eIF1A was able to rescue neuronal toxicity by enhancing translation in *Drosophila* (Moens et al., 2019). Ribosomal proteins are also found in a poly-GR mouse model and in cytoplasmic poly-GR and PR aggregates in patient tissue (Hartmann et al., 2018; Zhang et al., 2018b), suggesting that they may also sequester ribosomal proteins or assembled ribosomes as well. The arginine rich DPRs could also bind cellular RNAs rendering them insoluble and inaccessible to translation factors (Kanekura et al., 2016). An accumulation of nuclear mRNA has also been observed from expression of G_4_C_2_ repeats and proposed to be due to G_4_C_2_ repeat RNA sequestration of the nuclear export factor Aly/REF or the poly(A) binding protein PABP_C_ (Cooper-Knock et al., 2014; Rossi et al., 2015). Both the arginine rich DPRs and G_4_C_2_ repeat RNA also facilitate the phase separation of stress granules, whose role is to temporarily stall translation of sequestered mRNAs when required by the cell. Poly-GR and PR increase β-sheet content and enhance stability of stress granule protein LCD polymers, increasing the number of stress granules in a cell by reducing their dynamics and preventing disassembly (Lee et al., 2016; Lin et al., 2016; Boeynaems et al., 2017). Within G_4_C_2_ RNA-induced stress granules, the protein component G3BP1 maintained mobility whereas the repeat RNA did not (Fay et al., 2017), showing that alterations in phase separation can have varying impact on different constituents. Additionally, the C9orf72 protein, which is reduced in *C9orf72*-FTD/ALS, has been implicated in physiological stress granule formation (Maharjan et al., 2017), showing co-operative pathology from the *C9orf72* mutation. In addition, G_4_C_2_ repeat RNA has been identified in neuritic transport granules in murine and patient-derived neurons and in *Drosophila* where it colocalized with translational regulators and resulted in branching defects (Burguete et al., 2015). Thus, both DPR and G_4_C_2_ repeat RNA effects on the nucleolus, stress granules and other processes involved in translation can contribute to the translation repression seen in *C9orf72*-ALS/FTD.

#### Nucleocytoplasmic Transport

We have detailed above, how both the arginine rich DPRs and G_4_C_2_ repeat RNA can disrupt the nuclear pore. Poly-GR and poly-PR bind FG-domain containing nucleoporins, stabilizing their polymeric form, and thus may change the biophysical properties of the central channel (Shi et al., 2017). G_4_C_2_ repeat RNA can also lead to a loss of a selective group of nucleoporins with dysfunction of the FG-nucleoporin POM121 central to its pathogenic effect (Coyne et al., 2020), however the mechanism is yet unknown. In addition to nucleoporins, nucleocytoplasmic transport involves nuclear transport receptors and proteins involved in the Ran cycle. Further studies have provided evidence that the impact of the arginine rich DPRs on nucleocytoplasmic transport can also occur *via* transport receptor (also known as karyopherins) interaction. Poorly dynamic stress granules induced by expression of the arginine rich DPRs can sequester both nucleoporins and the transport receptors and thereby disrupt nucleocytoplasmic transport (Zhang et al., 2018a); indeed, transport receptors are known components of physiological stress granules (Chang and Tarn, 2009; Fujimura et al., 2010; Mahboubi et al., 2013). Transport receptor-mediated nuclear import is also impaired by the arginine rich DPRs (Jovičić et al., 2015; Solomon et al., 2018; Hayes et al., 2020; Cook et al., 2020; Hutten et al., 2020). The arginine rich DPRs bind several transport receptors *in vitro* (Lee et al., 2016; Hutten et al., 2020). Interaction with importin-β occurs *via* competition with the binding of arginine rich nuclear localization signals on cargo and thus inhibits their transport (Hayes et al., 2020), whereas polyPR-transportin-1 binding has recently been pinpointed to the nuclear localization recognition domain of transportin-1 (Nanaura et al., 2019, preprint). Arginine rich DPR binding reduces transport receptor solubility and drives their oligomerization and LLPS with transportin-1, importin-α or an importin-α/β complex (as occurs for physiological transport) being more susceptible than importin-β alone to these effects; exportin-1 remained unaffected in solubility and biophysical assays (Hutten et al., 2020). Condensates formed by poly-GR and transportin-1 showed no recovery after photobleaching showing that proteins are immobile in these structures. DPR binding was generally stronger for poly-GR than PR, which was reflected in the selective impairment of a classic NLS reporter or TDP-43 import by poly-GR. Disruption of transport receptor-dependent nucleocytoplasmic transport is however not observed in all studies, likely due to differences in assay sensitivities, transport factors or cell lines studied (Khosravi et al., 2017; Vanneste et al., 2019). Nucleocytoplasmic transport factors are also found with poly-GR, GA and GP aggregates in patient tissue (Khosravi et al., 2017; Solomon et al., 2018). Transport factors seem to be non-specifically prone to be sequestered into inclusions, as even those formed by artificial β-sheet containing proteins can induce this, and this process is specific to cytoplasmic and not nuclear accumulations (Woerner et al., 2016).

*C9orf72* G_4_C_2_ repeat RNA can also bind directly with nucleocytoplasmic transport factors, including Ran GTPase-activating protein 1 (RanGAP1; Donnelly et al., 2013). RanGAP is a key regulator of the Ran cycle which maintains the directionality of nucleocytoplasmic transport; it is anchored to the cytoplasmic face of the nuclear pore where it hydrolyzes RanGTP into RanGDP causing the dissociation of transport complexes releasing RanGDP and transport receptors to either release cargo into the cytoplasm for nuclear export or leave receptors available to bind cargo for import (Stewart, 2007). RanGAP overexpression was one of the strongest suppressors of toxicity from expression of 30 G_4_C_2_ repeats in a *Drosophila* screen and was found to mislocalize in patient brain tissue and in patient-derived neurons (Zhang et al., 2015), although the former finding in patient tissue has been disputed (Saberi et al., 2018). Cells expressing 30 G_4_C_2_ repeats and patient neurons also showed concomitant disturbances in the nuclear/cytoplasmic ratio of Ran, which could again be rescued by overexpression of RanGAP. Cytosolic accumulation of the *Drosophila* homolog of TDP-43, *TBPH*, was also found in *Drosophila* salivary gland cells expressing 30 G_4_C_2_ repeats, indicative of an imbalance in its nucleocytoplasmic shuttling. Although RanGAP does not contain any low complexity domains, interestingly its enzymatic activity can be dramatically enhanced when artificially targeted to liquid droplets (Peeples and Rosen, 2020, preprint). Noting that although the conclusions drawn implicate the *C9orf72* G_4_C_2_ repeat RNA, it is possible that effects were also mediated by low undetectable levels of DPRs translated from the RNA, as abnormalities in RanGAP distribution have been observed as a result of poly-PR expression in the cortex of mice (Zhang et al., 2019a). In summary, both DPRs and G_4_C_2_ repeat RNA can disrupt nucleocytoplasmic transport by affecting the nuclear pore directly, but also *via* transport receptors and the Ran cycle, much of which involves interactions with LCD containing proteins and alterations in phase separation behavior.

#### Genomic Homeostasis

Additional evidence also relates *C9orf72* pathogenesis and phase separation to genomic homeostasis. DNA damage has been associated with arginine rich DPR toxicity, in particular aberrant activation of the p53 pathway (Lopez-Gonzalez et al., 2016). Poly-PR has recently been shown to lead to a stabilization of p53 and the transcription of its targets, and p53 reduction or knockdown rescues both poly-GR and poly-PR toxicity in neurons and mice, and in G_4_C_2_ repeat expressing *Drosophila* and *C9orf72* patient-derived motor neurons (Maor-Nof et al., 2021). Interestingly, the nucleolus mediates the stabilization of p53 during DNA damage and regulates both its export and degradation (Rubbi and Milner, 2003; Boyd et al., 2011) and the nucleolar stress response pathway results in p53 accumulation (Rubbi and Milner, 2003; Yuan et al., 2005). As discussed above, both the arginine rich DPRs and G_4_C_2_ repeat RNA are associated with nucleolar dysfunction, with disruption in the phase separation of nucleolar proteins (White et al., 2019), and thus these pathologies may intersect in this pathway. In a mouse model expressing GFP-PR_50_ and in *C9orf72*-FTD/ALS patient tissue, poly-PR was found to colocalize with heterochromatin, highly condensed regions of chromatin which are transcriptionally inactive (Zhang et al., 2019a). These changes were not seen in GFP-(GR)_100_ expressing mice, likely due to the restricted cytoplasmic distribution of the DPR in this model, confirming that the actions of poly-PR are due to its nuclear localization. Poly-PR was subsequently found to disrupt the phase separation of heterochromatin protein 1α (HP1α) causing solid compartments to form within HP1α droplets and their bursting, and resulting in a significantly reduced number of droplets *in vitro*. This was recapitulated by reduced HP1α protein levels in the mouse model with functionally disrupted H3 histone post-translational modifications and upregulation of repetitive RNA elements, known to localize to heterochromatin, and double-stranded RNA which form from these which can initiate interferon signaling and cell death in neurons; these changes could also be induced by HP1α knockdown in cells. Interestingly, these changes occurred concurrently with irregularities in the nuclear lamina, which can also cause heterochromatin dysregulation (Scaffidi and Misteli, 2005; Shumaker et al., 2006). Indeed, changes in the nuclear lamina have been observed in *C9orf72*-FTD/ALS models (Zhang et al., 2019a), and loss of a *Drosophila* lamin enhanced *C9orf72* repeat toxicity (Freibaum et al., 2015). Thus, DPRs may also contribute to DNA damage and reduced transcription *via* alterations in genomic homeostasis.

### TDP-43 Aggregation and Phase Separation

Perturbed phase separation and membraneless organelle formation appears to explain one of the key pathological features of *C9orf72*-ALS/FTD, the nuclear depletion and cytoplasmic aggregation of TDP-43 present in 97% and 45% of ALS and FTD cases respectively (Ling et al., 2013). Unlike the widespread DPR and RNA foci pathology, TDP-43 pathology is highly correlated with brain areas showing the highest levels of neurodegeneration and clinical symptoms (Mackenzie et al., 2013; DeJesus-Hernandez et al., 2017). Based on this it has been proposed that TDP-43 mislocalization and/or aggregation are the most likely effectors of toxicity from the *C9orf72* mutation (Edbauer and Haass, 2016). Overt TDP-43 pathology is absent in many *in vivo* models of the *C9orf72* mutation (possibly due to the short lifespan of model organisms) but increased cytoplasmic accumulation, increased biochemical insolubility or phosphorylation have been observed in *Drosophila*, murine and cellular models expressing poly-GR and poly-GA (Khosravi et al., 2017, 2020; Schludi et al., 2017; Solomon et al., 2018; Cook et al., 2020; Hutten et al., 2020; LaClair et al., 2020; Park et al., 2020; West et al., 2020). There have also been reports of association between TDP-43 and DPR pathologies: dendritic-like aggregates of poly-GR co-localized almost completely with phosphorylated TDP-43 in *C9orf72*-ALS motor cortex but formed only a small proportion of these TDP-43 aggregates in total (Saberi et al., 2018); similarly, a proportion of both inclusions of poly-GR and poly-GA have been found to colocalize with TDP-43 in *C9orf72*-FTD/ALS hippocampus (Cook et al., 2020). In agreement with associations between these pathologies, the burden of TDP-43 and poly-GA, poly-GP or poly-GR inclusions correlates with neurodegeneration (Mackenzie et al., 2013, 2015; Gendron et al., 2015; Saberi et al., 2018; Sakae et al., 2018). Soluble poly-GP and poly-GR levels also correlate with clinical severity (Quaegebeur et al., 2020). Interestingly, there were also associations with the methylation status of poly-GR, which can affect its phase separation properties (Sakae et al., 2018; Gittings et al., 2020). *C9orf72* RNA foci composed of antisense C_4_G_2_ but not sense G_4_C_2_ transcripts have also been associated with the cytoplasmic mislocalization of TDP-43 in patient tissue (Cooper-Knock et al., 2015; Aladesuyi Arogundade et al., 2019).

**Figure 3:**
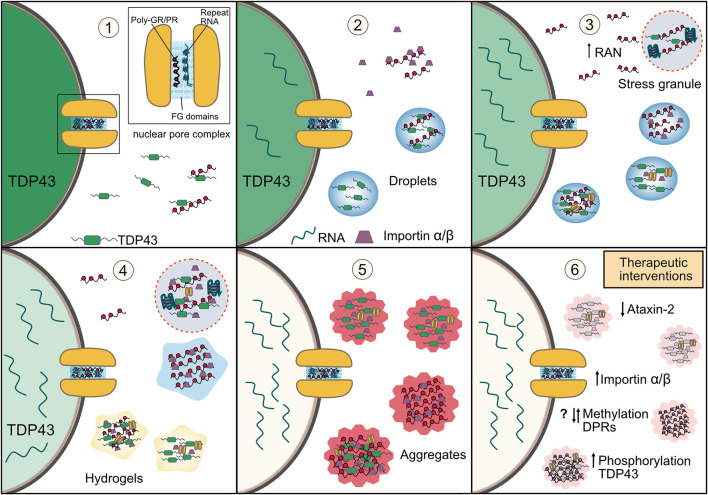
How disruptions in phase separation and membraneless organelles may lead to TDP-43 aggregation in *C9orf72*-ALS/FTD and possible therapeutic strategies. **(1)** *C9orf72* arginine rich DPRs and G_4_C_2_ repeat RNA bind nuclear pore proteins with phenylalanine rich repeats (FG domains) and result in nucleocytoplasmic transport dysfunction and mislocalization of TDP-43 to the cytoplasm. **(2)** Interaction between cytoplasmic TDP-43 and the arginine rich DPRs results in the LLPS of TDP-43 in the cytoplasm. Impaired nucleocytoplasmic transport also results in an accumulation of the importin-α/β complex (the import receptor for TDP-43) in the cytoplasm where it is also bound by the arginine rich DPRs and results in their reduced solubility. This begins a vicious feedback loop as impaired nuclear import of TDP-43 further increases levels of cytoplasmic TDP-43, whose LLPS is potentiated by a nuclear retention of mRNA from impaired nuclear export. **(3)** Cellular stress and the direct interaction of arginine rich DPRs and G_4_C_2_ RNA with stress granule proteins (including TDP-43) promotes phase separation and the formation of stress granules. The arginine rich DPRs also induce condensation of importin-α/β. TDP-43 droplets recruit importin-α/β complexes and nuclear pore proteins further impairing nucleocytoplasmic transport, resulting in more TDP-43 accumulation in the cytoplasm and depletion of nuclear TDP-43. Both stress granule accumulation and cytoplasmic TDP-43 also enhance RAN translation of the arginine rich DPRs. **(4)** The stress granules induced by the arginine rich DPRs and repeat RNA have reduced dynamics which entraps TDP-43, import receptors and nuclear pore proteins. Persistent TDP-43 and DPR-importin-α/β droplets are likely to mature into more solid-like states such as hydrogels, further immobilizing these proteins. **(5)** TDP-43 in solid-like states and within stress granules, and also DPRs, mature into pathological insoluble aggregates which further sequester proteins involved in nucleocytoplasmic transport. Thus, the disruption of phase separation and membraneless organelles leads to a cascade of vicious feedback loops which result in depletion of nuclear TDP-43 and its accumulation and aggregation in the cytoplasm in disease. **(6)** Therapeutic targeting of stress granules by reducing ataxin-2 levels, manipulating post-translation modifications such as methylation of the DPRs and phosphorylation of TDP-43, or increasing importin-α/β to reduce excessive LLPS may enhance the solubility of TDP-43, help to reduce its aggregation and ameliorate the pathological cascade in *C9orf72*-ALS/FTD and other TDP-43 proteinopathies.

Disrupted stress granule dynamics have been proposed to both directly and indirectly underlie the characteristic TDP-43 proteinopathy in ALS/FTD. Chronic stress can lead to an accumulation of TDP-43 in cytoplasmic stress granules, which become less dynamic by alterations in phase separation behavior from liquid to non-fluid gel states (Boeynaems and Gitler, 2018; McGurk et al., 2018b). Thus, the direct recruitment of TDP-43 to stress granules with impaired dynamics caused by the arginine rich DPRs and G_4_C_2_ repeat RNA may similarly explain pathological TDP-43 aggregation in DPR models and *C9orf72*-ALS/FTD. However, TDP-43 itself harbors an LCD and can also undergo LLPS *in vitro* (Molliex et al., 2015; Conicella et al., 2016, 2020; Schmidt et al., 2019) and form cytoplasmic droplets in cells independently of stress granules (Gasset-Rosa et al., 2019; Mann et al., 2019). RNA appears to be essential for these differences, with its presence being essential for recruitment of TDP-43 to stress granules and maintaining its solubility within them, and mutants lacking the ability to bind RNA forming immobile homomeric inclusions (Mann et al., 2019). This finding is consistent with TDP-43 inclusions in ALS/FTD patient tissue which do not contain stress granule proteins or RNA (Mann et al., 2019). Alterations in stress granule dynamics may thus indirectly prevent TDP-43 recruitment and promote homomeric TDP-43 inclusions. As the arginine rich DPRs and G_4_C_2_ repeat RNA perturb stress granule dynamics, their effect on TDP-43 aggregation may also similarly be indirect. A pathological retention of mRNA to poorly dynamic stress granules and DPR or repeat RNA-induced nuclear mRNA retention *via* impaired export (Freibaum et al., 2015; Rossi et al., 2015) would also further exacerbate this pathway by resulting in a cytoplasm deficient in mRNA preventing recruitment of TDP-43 to stress granules and promoting homomeric TDP-43 interactions in the cytoplasm resulting in further LLPS and the formation of more solid-like hydrogels of TDP-43 (Guenther et al., 2018) and its eventual aggregation. However, poly-GR interaction with TDP-43 does not require the RNA binding capability of TDP-43 (Cook et al., 2020) and can directly promote TDP-43 phase separation *in vitro* and reduce its solubility (Hutten et al., 2020). The direct effect of G_4_C_2_ RNA on TDP-43 phase separation has not yet been studied. In addition, DPR induced stress granule accumulation has been shown to specifically enhance RAN translation (Green et al., 2017; Cheng et al., 2018; Westergard et al., 2019), which would exacerbate any DPR induced mechanism discussed including cytoplasmic mislocalization of TDP-43; which itself has been shown to further enhance RAN translation (Solomon et al., 2018). Thus, both direct and indirect mechanisms relating to stress granules may account for pathological TDP-43 aggregation in DPR models and *C9orf72*-ALS/FTD (Figure 3).

In cells, although RNA-binding capacity was essential for TDP-43 aggregation, the formation of cytoplasmic inclusions (as predominates in disease) only occurred with a disrupted nuclear localization signal which localizes TDP-43 to the cytoplasm but not wildtype TDP-43 which remains in the nucleus (Mann et al., 2019). Similarly, TDP-43 recruitment to stress granules induced by poly-GR was also dependent on its prior cytoplasmic mislocalization with the same mutants (Cook et al., 2020). Together these indicate that nucleocytoplasmic transport pathology lies upstream of TDP-43 pathology, as has been previously suggested (Dormann and Haass, 2011; Boeynaems et al., 2016b). Described in detail above, both arginine rich DPRs and G_4_C_2_ RNA induced deficits in nucleocytoplasmic transport may occur through the sequestration or demixing of nucleocytoplasmic transport factors by inclusions of either DPRs or G_4_C_2_ RNA or DPR or RNA induced stress granules, or by direct disruption of FG nucleoporins in the nuclear pore, through which all transport occurs. Indeed, poly-PR induced mislocalization of nucleocytoplasmic transport factors was seen in the absence of TDP-43 pathology in a mouse model (Zhang et al., 2019a). It is currently unclear how a disruption in the nuclear pore structure or biophysical properties may lead to a directional imbalance in nucleocytoplasmic shuttling and cytoplasmic mislocalization of TDP-43. Cytoplasmic mislocalization of TDP-43 may be caused by either or both a reduction in nuclear import and enhancement of nuclear export. TDP-43 nuclear import is governed by active transport *via* importin-β binding to its nuclear localization signal, and thus reduced availability of transport receptors or Ran cycle proteins may have a major impact, whereas its export may either be passive or *via* redundant export receptors (Archbold et al., 2018; Ederle et al., 2018; Pinarbasi et al., 2018), and thus changes in the nuclear pore may have greater effect. Further both cytoplasmic TDP-43 (Solomon et al., 2018; Gasset-Rosa et al., 2019) and TDP-43 aggregates (Chou et al., 2018) can also sequester or lead to cytoplasmic demixing of proteins including nucleocytoplasmic transport factors which would again exacerbate these mechanisms– further enhancing TDP-43 mislocalization and aggregation. Together, through disruptions in phase separation behavior, stress granules and nucleocytoplasmic transport in combination with loss of nuclear TDP-43 autoregulation (Ayala et al., 2011; White et al., 2018), TDP-43 pathology may become independent of the initial DPR insult and maintain its own pathological cascade in a vicious feedback cycle (Solomon et al., 2018; Figure 3), which may explain the segregation of DPR and TDP-43 pathology in patient tissue.

### Therapeutics

We have described the evidence for the ability of the *C9orf72* arginine rich DPRs and G_4_C_2_ RNA to disrupt LLPS and perturb membraneless organelles leading to key pathomechanisms in disease, but what can be done to target these therapeutically to prevent TDP-43 aggregation and toxicity? One potential avenue would be to attempt to ameliorate the enhanced propensity for proteins to phase separate in the presence of pathological molecules and the resulting change in the biophysical properties and functionality of the droplets or organelles formed (Elbaum-Garfinkle, 2019). So far, the majority of treatments in this area relating to *C9orf72*-FTD/ALS and TDP-43 proteinopathy target stress granules. A high throughput screen identified a diverse range of small-molecule compounds that could alter stress granule properties; around half of these were compounds with planar moieties, such as Mitoxantrone which reduced both the size and number of stress granules formed, prevented recruitment of RNA-binding proteins (including TDP-43) to stress granules and decreased persistent TDP-43 puncta in cells treated with cellular stressors, and also ameliorated toxicity from overexpression of a mutant TDP-43 in primary neurons (Fang et al., 2019). Planar compounds intercalate into nucleic acids, and thus may act by preventing the RNA-dependent recruitment of TDP-43 to aberrant stress granules. Interestingly, another planar compound identified in this screen, doxorubicin, can alter the phase separation of CAG and CUG repeat RNAs and reduces G_4_C_2_ repeat RNA foci formation in cells (Jain and Vale, 2017). In the former screen, doxorubicin could also ameliorate toxicity from mutant TDP-43 but had differing cell line-dependent effects on stress granules and did not prevent the recruitment of TDP-43 (Fang et al., 2019). Thus, compounds with planar moieties act on multiple and varying mechanisms and maybe of therapeutic potential in *C9orf72*-FTD/ALS pathology. Another target of note is the stress granule protein ataxin-2, as its knockout or ASOs targeting ataxin-2 could significantly decrease TDP-43 aggregation and increase survival in a mouse model overexpressing wildtype TDP-43 (Becker et al., 2017). Ataxin-2 harbors two intrinsically disordered regions which both modulate phase separation behavior *in vitro* (Bakthavachalu et al., 2018). Ataxin-2 knockdown significantly decreases both the number of stress granules and the recruitment of endogenous TDP-43 (Becker et al., 2017). The arginine rich DPRs also directly interact with ataxin-2 (Lee et al., 2016; Hayes et al., 2020), colocalizing with poly-GR aggregates and TDP-43 in GR_200_ expressing mice (Cook et al., 2020). Knockdown of ataxin-2 can rescue mislocalization of Ran (part of the driving force for active nucleocytoplasmic transport) in *C9orf72* patient-derived neurons (Zhang et al., 2018a) and was the strongest suppressor of poly-GR toxicity in a *Drosophila* RNA inhibitor screen (Lee et al., 2016). This suppression could also be recapitulated when either intrinsically disordered region of ataxin-2 was deleted (Bakthavachalu et al., 2018), suggesting that its LLPS ability facilities *C9orf72* DPR toxicity. Hence it is possible that stress granule assembly promotes an environment for poly-GR to aggregate, undergo aberrant interactions (such as with TDP-43) and enhance toxicity, therefore modulating this process *via* reducing ataxin-2 levels may provide a novel therapeutic target in *C9orf72*-ALS/FTD (Figure 3, panel 6). Post translational modifications can also regulate the LLPS of proteins and the properties of the resulting condensates (Hofweber and Dormann, 2019) and thus could be targets for intervention. The post-translational modification poly-ADPribose (PAR) binds within the NLS of TDP-43 promoting its LLPS and is required for recruitment to stress granules (McGurk et al., 2018b). Knockdown of the PAR polymerases (PARPs) tankyrase 1/2 or small molecule inhibition of PARP-1/2 could both reduce toxicity from overexpression of TDP-43 in *Drosophila* or spinal cord cultures, respectively (McGurk et al., 2018a, b). Interestingly, the small molecule inhibitors of PARP-1/2 reduce the formation of TDP-43-positive stress granules in cells (McGurk et al., 2018a), whereas the tankyrase-1/2 inhibitors do not affect stress granules but prevent TDP-43 recruitment to them (McGurk et al., 2018b), suggesting that the latter may act more specifically on TDP-43. Indeed, recent data demonstrates a novel binding site for the tankyrases on TDP-43 and suggests that binding may prevent its ubiquitination and proteasomal turnover in the nucleus, stabilizing TDP-43 in the cytoplasm (McGurk et al., 2020). Thus, either direct or indirect pathways associated with LLPS and stress granules are showing promise as therapies for *C9orf72-FTD/ALS*.

Targeting DPRs or TDP-43 directly to prevent their aberrant phase separation and aggregation may also be effective. It has been recently shown that anti-cancer drugs partition into droplets formed by their molecular targets and change the properties of the condensate (Klein et al., 2020). Two molecules which have recently shown promise for ALS are lipoamide and lipoic acid which prevent FUS aggregation *via* influencing FUS phase separation and reduce FUS toxicity *in vivo* (Wheeler et al., 2019). The identification of small molecules and compounds that can modulate the charge-charge and cation-pi interactions essential for phase separation of LCD containing proteins is an interesting avenue for future research, although a greater understanding is needed into the physiochemical mechanisms as to how molecules such as lipoamide and lipoic acid disrupt these interactions inhibiting LLPS (Wheeler, 2020). Again, post-translational modifications can be targeted. As detailed above symmetric dimethylation of poly-GR reduces its propensity for LLPS and appears to be protective (Gittings et al., 2020; Pakravan et al., 2020). The enzymes responsible for arginine methylation belong to the protein arginine methyltransferase (PRMT) family (Yang and Bedford, 2013). Knockdown of several PRMTs enhance toxicity in *Drosophila* models of poly-PR toxicity, with PRMT1 also colocalizing with poly-PR upon co-transfection in cell lines (Boeynaems et al., 2016a), in agreement with the protective role suggested by human studies. However, small molecule inhibition of type I PRMTs, which produce asymmetrically dimethylated arginine, alleviated the toxicity of both poly-GR and PR in cells (Premasiri et al., 2020). This could align with the human studies as symmetric dimethylation would protect the arginine residues from asymmetric demethylation, although it does not explain why knockdown of the type I PRMT PRMT1 exacerbated toxicity in the *Drosophila* studies. These opposing roles do not however align with their effect on LLPS, as both symmetric and asymmetric demethylation of poly-GR reduce propensity to undergo LLPS. Interestingly, a rapid demethylation of the stress granule protein G3BP1 is critical for stress granule assembly (Tsai et al., 2016), showing that methylation can modulate LLPS behavior in different contexts and that the process can also be dynamic. Further work will be required to assimilate these findings and determine whether modulating the phase separation behavior of the arginine rich DPRs by altering their methylation state is a valid therapeutic target (Figure 3, panel 6). Phosphorylation can also both enhance and hinder LLPS of ribonuclear granules *in vitro* (Hofweber et al., 2018). In the context of TDP-43, phosphorylation of its N-terminal domain inhibits LLPS (Wang et al., 2018a) and C-terminal domain phosphorylation has been shown to reduce TDP-43 aggregation and toxicity in cells (Li et al., 2011), suggesting that the associated kinases or phosphatases could be targeted (Figure 3, panel 6). However, as is commonly found in neurodegeneration (Hofweber and Dormann, 2019), TDP-43 inclusions are hyperphosphorylated in patient brain which may suggest that a role in aggregate formation (Hasegawa et al., 2008). Also, post-translation modifications regulate a vast number of protein and cellular functions, hence targeting them on a specific molecule of interest is incredibly challenging and complicating this even further is the fact numerous post-translational modification enzymes have been associated with ALS (Guo et al., 2020).

It has also been proposed that chaperones exist whose normal function is to modulate LLPS within a cellular environment that is particularly concentrated, unstable and oversaturated in order to prevent abnormal fibrillization (Elbaum-Garfinkle, 2019). Such chaperones may provide more specific therapeutic targets, rather than modifying the LLPS of membraneless organelles or post-translational modifications which are both vitally important in normal cell function. Heat shock proteins are a classic example of cellular chaperones and the master transcriptional regulator of heat shock protein expression HSF1 can prevent cytoplasmic accumulation and toxicity from over-expression of wildtype or mutant TDP-43; the associated reduction in TDP-43 solubility was found to be enacted by the heat shock protein DNAJB2a (Chen et al., 2016). Variants of the heat shock protein Hsp104 engineered to potentiate disaggregation are also able to dissolve both FUS and TDP-43 aggregates in yeast (Jackrel and Shorter, 2014; Jackrel et al., 2014) and FUS aggregates in cells (Yasuda et al., 2017). Interestingly, nuclear import receptors, associated with both DPR and TDP-43 toxicity in models and post-mortem brain (Nishimura et al., 2010; Jovičić et al., 2015; Solomon et al., 2018; Gasset-Rosa et al., 2019; Hayes et al., 2020; Cook et al., 2020; Hutten et al., 2020; Park et al., 2020) have also been shown to prevent the pathological phase separation of RNA binding proteins (Springhower et al., 2020). Transportin-1 can both prevent phase separation, hydrogel formation and fibrillization of FUS and hnRNP A1 and A2, and importantly dissolve already formed hydrogels and fibrillar aggregates (Guo et al., 2018; Qamar et al., 2018; Yoshizawa et al., 2018; Hofweber et al., 2018). ALS-associated mutations in the NLS of FUS disrupt binding to transportin-1, preventing its solubilizing effects on FUS and leading to FUS accumulation in stress granules (Hofweber et al., 2018). Functionally, increasing transportin-1 expression could also restore nuclear localization of FUS or hnRNPA1/2, restore proteins synthesis in hypomethylated FUS neurons, and improve lifespan and muscle degeneration phenotypes in *Drosophila* models of mutant FUS and hnRNP A2, respectively (Guo et al., 2018; Qamar et al., 2018). Similarly, an importin-α/β complex reduces *in vitro* LLPS and fibril formation of TDP-43 and could also disassemble preformed fibrils (Guo et al., 2018). TDP-43 is transported into the nucleus *via* its classic NLS recognized by the importin-α/β complex, whilst FUS and the hnRNPs contain a PY-NLS which is bound and transported by transportin-1 (Mihevc et al., 2017). If the PY-NLS of FUS is substituted for a classic NLS, the importin-α/β complex can now inhibit its phase separation (Yoshizawa et al., 2018). Hence, nuclear import receptors modulate the phase separation and fibril formation of cargo in their respective nucleocytoplasmic transport pathway, indicating an additional physiological chaperone function of transport receptors in maintaining solubility of their cargo. Thus, potential disease therapies will likely need to reflect the major pathology involved. In the case of *C9orf72*-ALS/FTD where TDP-43 is the major pathology, the arginine rich DPRs bind to importin-α and β, reducing their solubility and lead to their precipitation and condensation; this results in defective nuclear import of TDP-43, and thus likely contributes to the dominant TDP-43 pathology (Hutten et al., 2020). High concentrations of importin-α/β can suppress poly-GR induced TDP-43 phase separation *in vitro* and its reduced solubility in cells, and inhibit RNA-driven LLPS of poly-GR. Therefore, therapies boosting levels of importin-α and β could ameliorate both DPR and TDP-43 pathologies and prevent other pathogenic interactions, which may be beneficial for the wide range of cellular abnormalities observed in *C9orf72*-ALS/FTD (Figure 3, panel 6). Notably, both heat shock proteins and import receptors are reduced in ALS/FTD patient tissue (Kinoshita et al., 2009; Nishimura et al., 2010; Chen et al., 2016; Solomon et al., 2018), and thus supplementation strategies will also restore physiological roles lost in disease.

A complementary strategy to all of the above strategies is to target the *C9orf72* G_4_C_2_ repeat RNA and DPRs directly to reduce their levels and prevent pathological intra and intermolecular associations. An approach that has gained much traction and is being pursued in clinical trials is using ASOs to the *C9orf72* repeat which can reduce the formation of repeat RNA and DPRs in *C9orf72* repeat-expressing neurons and mice and patient-derived neurons with an associated reduction in glutamate-induced toxicity in cultured neurons and behavioral and cognitive phenotypes in mice (Donnelly et al., 2013; Lagier-Tourenne et al., 2013; Sareen et al., 2013; Jiang et al., 2016; Gendron et al., 2017). Small molecules which bind G_4_C_2_ G-quadruplexes can reduce the production of DPRs and rescue toxicity (Zhang et al., 2015; Simone et al., 2018), potentially by destabilizing and ablating G-quadruplex dependent interactions (Zamiri et al., 2014), which may prevent its nucleation behavior in relation to RNA-dependent protein condensation and stress granule formation discussed above. Endogenous targets that modulate the production of repeat RNA and/or RAN translation have also been identified. Reducing the transcription elongation factor Spt4 or PAF1C complex, a transcriptional regulator of RNA polymerase II, reduce production of expanded G_4_C_2_ repeat RNA and thus the translation of DPRs and their associated toxicity in model systems (Liu et al., 2012; Kramer et al., 2016; Goodman et al., 2019a). Conversely, the RNA-binding protein hnRNPA3 can bind G_4_C_2_ repeat RNA and its depletion increases production of repeat RNA and DPRs (Mori et al., 2013b, 2016). Genetic screens have also identified regulators of RAN translation, including the small ribosomal protein RPS25 and the eukaryotic translation initiation factors eIF4B and eIF4H, whose loss could reduce the production of DPRs and extend lifespan in *Drosophila* models and the former also in *C9orf72* patient cells (Goodman et al., 2019b; Yamada et al., 2019). The integrated stress response also promotes RAN translation (Green et al., 2017; Cheng et al., 2018; Westergard et al., 2019), and inhibition of one part of this—the double-stranded RNA-dependent protein kinase (PKR) pathway—can reduce RAN translation and mitigate gliosis, motor neuron loss and behavioral phenotypes in a *C9orf72* mutation mouse model (Zu et al., 2020). Of note, hnRNPA3 and eIF4H are reduced in *C9orf72*-FTD/ALS patient brain (Mori et al., 2016), and therefore supplementation may provide therapeutic benefit, whereas components of the PAF1C complex are upregulated and the PKR pathway is aberrantly activated in disease and thus inhibition of these pathways may normalize levels (Goodman et al., 2019a; Zu et al., 2020).

## Summary

There is now substantial and clear evidence that a disruption in the phase separation behavior of proteins and RNA involved in the formation of liquid-like membraneless organelles explains much of the major pathological phenomena associated with *C9orf72*-ALS/FTD. Gain-of-function mechanisms associated with the G_4_C_2_ repeat expansion in *C9orf72*–G_4_C_2_ repeat RNA and the arginine rich DPRs poly-GR and poly-PR—undergo phase separation themselves and perturb the phase separation of LCD containing proteins, resulting in abnormal membraneless organelle formation and dissolution, impairing their physiological functions and leading to neurodegeneration. Further pathological phase separation induced by the arginine rich DPRs is strongly associated with TDP-43 dysfunction and aggregation, the major pathological hallmark of *C9orf72-ALS/FTD* correlating with neuronal cell death. The targeting of abnormally phase separated condensates using small molecules or gene therapy provides a novel strategy for future therapeutics, although a greater understanding is needed of phase separation in order to design targets which are both beneficial and precise.

## Author Contributions

DS and SM conceived the review. DS, RS and SM wrote sections of the manuscript. MR and DS designed and produced figures. All authors contributed to the article and approved the submitted version.

## Conflict of Interest

The authors declare that the research was conducted in the absence of any commercial or financial relationships that could be construed as a potential conflict of interest.
